# Unbiased In Silico Analysis of Gene Expression Pinpoints Circulating miRNAs Targeting *KIAA1324*, a New Gene Drastically Downregulated in Ovarian Endometriosis

**DOI:** 10.3390/biomedicines10092065

**Published:** 2022-08-24

**Authors:** Carole Abo, Louise Biquard, Laura Girardet, Sandrine Chouzenoux, Pierre-Alexandre Just, Charles Chapron, Daniel Vaiman, Bruno Borghese

**Affiliations:** 1 U1016 Institut Cochin, Institut National de la Santé et de la Recherche Médicale, UMR8104 Centre National de la Recherche Scientifique, 75016 Paris, France; 2 Faculty of Medicine, University of Paris, 75006 Paris, France; 3 Department of Pathological Anatomy and Cytology, Hôpital Cochin, Assistance Publique—Hôpitaux de Paris, 75004 Paris, France; 4 Department of Gynecologic Surgery, Hôpital Cochin, Assistance Publique—Hôpitaux de Paris, 75004 Paris, France

**Keywords:** endometriosis, miRNA, biomarkers, KIAA1324 (EIG121)

## Abstract

Objective: To identify circulating miRNAs associated with ovarian endometriosis (OMA), and to analyze candidate genes targeted by these miRNAs. Methods: Putative regulating miRNAs were identified through an original bioinformatics approach. We first queried the miRWalk 2.0 database to collect putative miRNA targets. Then, we matched it to a transcriptomic dataset of OMA. Moving from gene expression in the tissue to possible alterations in the patient plasma, a selection of these miRNAs was quantified by qRT-PCR in plasma samples from 93 patients with isolated OMA and 95 patients surgically checked as free from endometriosis. Then, we characterized the genes regulated by more than one miRNA and validated them by immunohistochemistry and transfection experiments on endometrial cell primary cultures obtained from endometrial biopsies of 10 women with and without endometriosis with miRNA mimics. Stromal and epithelial cells were isolated and cultured separately and gene expression levels were measured by RT-qPCR. Results: Eight miRNAs were identified by bioinformatics analysis. Two of them were overexpressed in plasma from OMA patients: *let-7b-5p* and *miR-92a-3p* (*p* < 0.005). Three miRNAs, *let-7b* and *miR-92a-3p*, and *miR-93-5p* potentially targeted *KIAA1324*, an estrogen-responsive gene and one of the most downregulated genes in OMA. Transfection experiments with mimics of these two miRNAs showed a strong decrease in *KIAA1324* expression, up to 40%. Immunohistochemistry revealed a moderate-to-intense staining for KIAA1324 in the eutopic endometrium and a faint-to-moderate staining in the ectopic endometrium for half of the samples, which is concordant with the transcriptomic data. Discussion and Conclusion: Our results suggested that *KIAA1324* might be involved in endometriosis through the downregulating action of two circulating miRNAs. As these miRNAs were found to be overexpressed, their quantification in plasma could provide a tool for an early diagnosis of endometriosis.

## 1. Introduction

Endometriosis is a frequent gynecologic disease inducing a chronic inflammatory state [1]. Interest in endometriosis has substantially increased over the last couple of years, in regard to its distinctive features: (i) its rather common but non-specific symptoms, including chronic pain and infertility, (ii) its association with many diseases, mostly inflammatory or auto-immune, and (iii) its significant impact on the quality of life [2].

Management of endometriosis has recently evolved. Hormonal and non-hormonal treatments combined with assisted reproductive technologies and surgery when indicated are established as the first-line options, whereas incomplete and repetitive surgeries have been set aside [2]. Imaging techniques have drastically improved over the past few years for diagnosing endometriosis, but they are merely useful for phenotyping the disease and distinguishing the well-known subtypes: superficial peritoneal endometriosis, ovarian endometrioma and deeply infiltrating endometriosis [3,4,5,6]. Yet, the diagnosis of endometriosis remains unsatisfactory. Even today, it may take many years to establish an accurate diagnosis [7], partly due to the poor knowledge of the disease by physicians, and partly due to the existence of superficial forms that can be undetected, even by magnetic resonance imaging [6]. Therefore, making an early and accurate diagnosis is extremely important, as it allows physicians to propose an appropriate and patient-centered approach, based on the following principles: (i) treat the pain effectively and as soon as possible; and (ii) plan the future with a lifetime perspective, considering the patient’s wishes (for instance, desire for pregnancy or not) and a range of options that are not mutually exclusive (for instance, fertility preservation, use of oral contraceptives, “one-shot” surgery, etc.) [2].

Circulating biomarkers of endometriosis could theoretically contribute to shortening the time to diagnosis, to refine this diagnosis and to contribute to the understanding of the disease. As such, microRNAs (miRNAs) have gained a great interest in the field of endometriosis. In various contexts, miRNA can regulate proliferation, inflammation, angiogenesis, tissue repair or extracellular matrix remodeling, or other processes implicated in the development of endometriosis [8,9]. In our recent review on the epigenetics of endometriosis, we showed that data published on miRNAs were rather scarce and conflicting, probably because of the heterogeneity of the disease and the existence of several subtypes that have not been properly taken into consideration in the analyses so far [10].

The aim of the present study is to identify miRNAs potentially involved in the disease, using an unbiased bioinformatics approach without preconceived hypothesis. First, we decided to focus on a well-defined subtype of endometriosis: the isolated ovarian endometrioma (OMA), which is a subtype of endometriosis without the associated deep endometriotic nodules. Then, we chose an original design for the study, based on the following steps: (i) collect transcriptomic data regarding the most differentially expressed genes in OMA when compared to the eutopic endometrium; (ii) identify putative miRNAs that could target those genes by bioinformatic analysis; (iii) quantify those putative miRNAs, first in the ectopic endometrium, then in plasma, from patients with OMA; and (iv) characterize the genes regulated by more than one miRNA, and validate them by immunohistochemistry, Western blot and transfection experiments on endometrial cell primary cultures. More precisely, we selected *KIAA1324*, one of the most downregulated genes in OMA [11] to evaluate what mechanistical part is played by specific miRNAs on the regulation of this specific gene. *KIAA1324* is now also known as *EIG121* (Estrogen Induced Gene 121) and *ELAPOR1* (endosome-lysosome associated apoptosis and autophagy regulator family protein-1), underlining the connections of this gene with two major features of OMA (estrogen-dependence) and autophagy regulation in the context of cell growth [12].

## 2. Materials and Methods

### 2.1. Study Population and Sample Collection

The study included 216 women of reproductive age, aged 41 or below, who underwent a laparoscopic surgery in our tertiary referral center (Table 1). A total of 113 patients with OMA (the endometriosis group) and 103 patients without endometriosis (the control group) were enrolled for further experiments. Individuals of the control group were operated on for a benign gynecologic condition (mostly infertility or a benign ovarian cyst) and endometriosis was ruled out during the surgery by a thorough examination of the peritoneal cavity and/or after the pathologic examination of the ovarian cyst that excluded an OMA. Patients in the endometriosis group had an isolated OMA without deep nodules, as checked preoperatively by MRI and during the surgery by a careful exploration of the abdomino-pelvic cavity. The diagnosis of OMA was systematically confirmed by an expert pathologist (P-A.J.).

Clinical characteristics of the patients were recorded before surgery using standardized questionnaires that were previously described [13]. The phase of the menstrual cycle (proliferative or secretory) was noted at the time of surgery and confirmed histologically by the Noyes criteria for the cases where endometrial samples were available. The study was approved by the local ethics committee (Comité de Protection des Personnes Paris-Cochin Nr. 05-2006). All patients signed an informed consent to participate in this study.

Plasma samples from 93 patients with OMA and 95 controls were collected in the operating room, just before the beginning of the surgical procedure. After insertion of the peripheral venous catheter, 5–10 mL of venous blood samples were collected into EDTA tubes, centrifuged at 2000 rpm for 12 min at 4 °C. Plasma supernatants were collected, and aliquots of those samples were stored at −70 °C until use.

Tissue samples from 20 patients with OMA (a piece of the cyst wall and fragments of the eutopic endometrium) and 8 controls (fragments of the normal endometrium) were processed and immediately frozen into liquid nitrogen in the operative room and then stored at −80 °C until use. A set of them was processed for Western blot (4 samples), primary endometrial culture (10 samples) and formalin fixation and paraffin embedding (6 samples) for further transfection experiments and immunohistochemistry assays, respectively (Table 2 and Table 3).

### 2.2. Computer-Assisted Analysis, RNA Extraction and qPCR Analyses

Bioinformatic analyses were conducted on a transcriptomic dataset regarding gene expression in OMA, which has been previously published [11]. We referred to the miRWalk2.0 database (free access on: http://mirwalk.umm.uni-heidelberg.de, accessed on 1 January 2019), that represents a comprehensive atlas of all predicted and validated miRNA-target interactions [14]. We queried the miRWalk database to obtain a list of all known miRNA and, for each of them, the genes that they regulate. We crossed this database with the transcriptomic dataset of the genes differentially expressed in OMA when compared to the eutopic endometrium. In the latter dataset, we considered the genes with an induction ratio above 2.0 as upregulated and below 0.5 as downregulated. Concretely, for a given miRNA we tested by means of a chi-square test of contingency, whereas all of the genes regulated by this miRNA had an expressional bias in the OMA when compared to a set of genes randomly chosen from the whole genome. The most significant miRNAs were considered for further analysis. Thus, we were able to unveil which miRNAs were likely to be at play in endometriosis.

Total RNA was extracted from tissue samples (normal endometrium for the controls, eutopic endometrium and OMA for the cases) using TRIzol reagent (Invitrogen, Carlsbad, CA, USA), according to the manufacturer’s instructions. RNA was treated with DNAse (Invitrogen) to remove any contaminating DNA. Total RNA was extracted from the plasma by means of the Nucleospin miRNA Plasma Kit (Macherey Nagel) according to the manufacturer’s protocol and was eluted in nuclease-free water. RNA samples were analyzed with a Nanodrop ND 2000 spectrophotometer. Complementary DNA (cDNA) was synthetized using reverse transcription. We used the TaqMan^®^ Advanced miRNA cDNA synthesis Kit (ThermoFisher, Les Ulis, France) according to the manufacturer’s instructions. Briefly, the total RNA from each sample was reverse-transcribed. Then, the expression level of miRNAs was assessed using Taqman^®^ Advanced miRNA Assays kits; one specific kit was used for each considered miRNA (as detailed in the Supplemental Methods) (*miR-484*: Assay ID 478308; *miR-192-5p*: Assay ID 478262; *miR-16-5p*: Assay ID 477860; *miR-215-5p*: Assay ID 478516; *let-7b-5p* Assay ID 478576; *miR-92a-3p*: Assay ID 477827; *miR-93-5p*: Assay ID 478210; and *miR-30a-5p*: Assay ID 479448), and TaqMan^®^ Fast Advanced Master Mix was used according to the manufacturer’s protocol. The reaction mixture included 5 μL of TaqMan^®^ Fast Advanced Master Mix, 1 μL of TaqMan^®^ Advanced miRNA Assay (specific for each miRNA), 4 μL of RNAse-free water and 5 μL of cDNA. Quantitative Real-Time Polymerase Chain Reaction (qPCR) was performed in a LightCycler^®^ 480 Instrument II (Roche Life Science, Penzberg, Germany). The qPCR protocol consisted of an initial denaturation of 5 min at 95 °C followed by 40 cycles of 10 s at 95 °C, annealing at 60 °C for 20 s, 72 °C for 10 s and a final melting curve. The relative miRNA expression was determined by the 2-ΔΔCt method. U6 small nuclear RNA was used as a control to evaluate the relative miRNA level. Primers were obtained from Eurogentec (Angers, France) (*miR-484*: Assay ID 478308, UCAGGCUCAGUCCCCUCCCGAU; *miR-192-5p*: Assay ID 478262, CUGACCUAUGAAUUGACAGCC; *miR-16-5p*: Assay ID 477860, UAGCAGCACGUAAAUAUUGGCG; *miR-215-5p*: Assay ID 478516, AUGACCUAUGAAUUGACAGAC; *let-7b-5p*: Assay ID 478576, UGAGGUAGUAGGUUGUGUGGUU; *miR-92a-3p*: Assay ID 477827, UAUUGCACUUGUCCCGGCCUGU; *miR-93-5p*: Assay ID 478210, CAAAGUGCUGUUCGUGCAGGUAG; *miR-30a-5p*: Assay ID 479448, UGUAAACAUCCUCGACUGGAAG; and *U6*: forward CTCGCTTCGGCAGCACA, reverse AACGCTTCACGAATTTGCGT). The results were analyzed with the LightCycler Software using the three fit point method.

### 2.3. Isolation and Primary Culture of Endometrial Stromal and Epithelial Cells

Primary endometrial cell cultures were prepared from eutopic endometrial biopsies as well as from lesions (ectopic endometrium), according to our previously published protocol [15]. Biopsy specimens were rinsed and minced into small pieces and then digested with dispase and collagenase (2 mg/mL, Gibco Invitrogen, Cergy Pontoise, France) for 1h at 37 °C and separated using serial filtration. Red blood cells were removed by hypotonic lysis (using 0.15M NH_4_Cl, 1mM KHCO_3_ and 0.1mM Na_2_ EDTA). Debris was removed using sieves with 100 μm apertures; the epithelial cells were retained on sieves with 100 μm apertures while the stromal cells remained in the filtrate. Both of these cell types were plated onto Primaria flasks (Becton Dickinson Labware, Le Pont de Claix, France) and cultured in Dulbecco’s modified Eagle’s medium (Gibco Invitrogen, Cergy Pontoise, France) with 10% fetal calf serum. For each sample, two cell populations were obtained: stromal cells and epithelial cells. The purity of the stromal and the epithelial cell suspensions was assessed by staining with a 1:100 dilution of fluorescein-isothiocyanate-labelled anti-cytokeratin and Cy3-labelled anti-vimentin antibodies, respectively (Sigma-Aldrich, St Louis, MI, USA). The fluorescence signal was imaged using an Olympus fluorescent microscope (Hamburg, Germany) and images were captured using the CellM Imaging station (Olympus). Both of the cell populations were negative for CD3 (T cells), CD45 (leucocytes) and CD11b (monocytes and granulocytes) staining. The cell samples were only used when they were at least 90% pure. Each cell type was cultured in its specific medium until they reached 90% confluence, which occurred between 7 and 21 days after the collection of the samples.

### 2.4. Cell transfection, Total RNA Extraction and qPCR for KIAA1324 Gene

The cells were cultured on 24-well plates to confluency and transfected with miRVana™ miRNA Mimics (Thermo Fischer Scientific, Les Ulis France) that are small, chemically modified double-stranded RNAs that mimic endogenous miRNA: *hsa-let-7b-5p* mimic (MC11050), *has-miR-92a-3p* (MC10916), and both of them, or a control siRNA-A (Santa Cruz Biotechnology) as a negative control. Transfection was performed using Lipofectamine 2000 (Invitrogen) according to the manufacturer’s protocol. After 24 h, a hormonal treatment was added with 17-β estradiol (Sigma-Aldrich Burlington, USA) in concentrations of 10^−6^ M, 10^−8^ M or 0 M, diluted in ethanol and supplemented to serum-free medium. The transfected cells were harvested 24 h after hormonal treatment. Total RNA was extracted from the cultured cells using TRIzol reagent (Invitrogen, Carlsbad, CA, USA), according to the manufacturer’s instructions. Each well was processed separately. RNA was treated with DNAse (Invitrogen) to remove any contaminating DNA. RNA concentration was determined using a Nanodrop ND 2000 spectrophotometer. One microgram of total RNA was reverse-transcribed with random primers and M-MLV Reverse Transcriptase (Invitrogen, Les Ulis France), according to the manufacturer’s protocol. The relative quantity of *KIAA1324* cDNA was assessed by qPCR. Primers were used at 10 nM in the PCR reaction. qPCR was carried out on a LightCycler^®^ 480, 96-well apparatus (Roche Diagnostics, Manheim, Germany) with the use of the SYBR Green I Master (Roche Diagnostics, Manheim, Germany), according to the manufacturer’s instructions. The thermal cycling conditions consisted of an initial denaturation of 5 min at 95 °C followed by 40 cycles of 10 s at 95 °C, annealing at 60 °C for 20 s, 72 °C for 10 s and a final melting curve. The relative expression of the target gene normalized with an internal control gene (Cyclophilin, Peptidyl-prolyl cis-trans isomerase B or *PPIB*) was presented using the 2^−ΔΔCt^ method. The results were analyzed with the LightCycler Software using the three fit point method. Primers for qPCR were designed using the PRIMER3 software, based on published sequences and were synthesized by Eurogentec (Angers, France): *KIAA1324*: forward CAGGGCTCCTCTTTCTGCAA, reverse AGTTGTGTCTCTCCGTTGGC, *PPIB*: forward AAGTCACCGTCAAGGTGTATTTT, reverse TGCTGTTTTTGTAGCCAAATCCT.

### 2.5. Immunohistochemistry and Western Blot for KIAA1234 Protein

For each endometriotic patient, we included one slide of the eutopic endometrium and one slide of OMA. Immunohistochemistry was performed using a polyclonal antibody against the KIAA1324 protein (the COOH-terminal peptide) (Dilution: 1/100) designed by Sigma Genosys (Houston, TX, USA). This antibody was a kind gift from Dr. Russell R. Broaddus from the Department of Pathology, University of Texas, M. D Anderson Cancer Center and validated by their study [16]. Briefly, 5 μm sections were cut sequentially and mounted onto super-frost-treated slides (Menzel-Glasse, Braunschweig, Germany). The slides were dried overnight at 37 °C before deparaffinization in xylene and rehydratation through graded ethanols. For epitope retrieval, the slides were immersed in a water bath at 96 °C for 90 min with a citrate buffer, at pH 6.0 (S236, Dako Corp, Glostrup, Denmark). Then, the slides were cooled in their buffer for 20 min at room temperature. H_2_O_2_ (0.3%) was added to the slides and the slides were incubated at room temperature for 30 min. The tissues were then incubated for 2 h with the primary antibodies. The NovolLink max Polymer Detection System (Newcastle, UK) was used for the subsequent steps, according to the manufacturer’s instructions. Chromogenic development was accomplished using diaminobenzidine-hydrogen peroxide. The slides were then slightly counterstained with hematoxylin and dehydrated, and a coverslip was applied. Since the antibody was initially developed for the pancreas, we also tested it in this tissue (Supplementary Figure S1). Immunohistochemistry was conducted by an expert pathologist in the field of endometriosis (P.-A.J.).

### 2.6. Statistical Analysis

All data were collected in a computerized database and analyzed with the software Prism 6 (GraphPad Software, Inc., San Diego, CA, USA). Parametric statistics were used for normally distributed data. We used a Student’s *t*-test for quantitative variables, Pearson’s chi-square and Fisher’s exact tests for qualitative variables and chi-square for the detection of the relevant miRNAs, as appropriate. A one-way ANOVA was conducted when more than two groups were compared. Non-parametric statistics were used for non-normally distributed data. When more than two groups were compared, we used a Kruskal–Wallis test. When group medians were significantly different compared with the multigroup tests (*p* < 0.05), pairwise comparisons were performed using Dunn’s Multiple Comparison Test. The correlation analysis of continuous variables was based on the Spearman test correlation method. The diagnostic performance (sensitivity and specificity) of the plasma miRNA expression levels for endometriosis was estimated with the receiver operating characteristic (ROC) curve, and the area under the ROC curve was calculated. In every figure, the error bars represent the standard error of the mean and *p* < 0.05 was considered significant.

## 3. Results

### 3.1. Clinical Characteristics of the Patients

The study included 113 patients surgically diagnosed with isolated OMA (the endometriosis group, without additional lesions in other places) and 103 patients with no evidence of the disease, as checked unambiguously by a thorough surgical exploration of the peritoneal cavity (the control group). The main indications for surgery in the control group were a benign ovarian cyst (except OMA) (43%) and an infertility workup (57%). The main characteristics of the two groups are presented as Table 1. We observed a higher proportion of current use of hormonal treatment in the control group than in the endometriosis group (58% versus 19%, *p* = 0.002). However, there was a large amount of missing data for this parameter; this did not allow us to provide valid statistics for this parameter. As expected, patients with endometriosis were in more pain than the controls, especially for dysmenorrhea and deep dyspareunia (*p* = 0.0007 and 0.01, respectively). The infertility rate was marginally significant in secondary infertilities (*p* = 0.018). There was an equal proportion of patients in the follicular and secretory phases in both groups.

### 3.2. Bioinformatic Identification of miRNAs Associated with Endometrioma

The aim of this task was to predict which miRNAs could modulate endometriosis development. We used the miRWalk2.0 database to retrieve all known miRNAs and, for each of them, the genes that they regulate. We crossed the data provided by miR-Walk2.0 with the transcriptomic data obtained previously on OMA [11]. We tested, by means of a chi-square test of contingency, whether the set of genes regulated by a given miRNA presented an expressional bias according to the transcriptomic data in OMA when compared with a random set of genes taken from the whole transcriptome. In this way, we were able to determine, with an objective approach using global data-bases, which miRNAs could have a role in endometriosis-driven gene deregulations. Table 4 shows the results of the bioinformatic analysis as a list of the 24-most-significant miRNAs.

Among these 24 miRNAs, one third (*n* = 8) was previously cited in publications on endometriosis: *miR-16-5p*, *let-7b-5p*, *miR-93-5p*, *miR-149-5p*, *miR-222-3p*, *miR-93-3p*, *miR-1* and *miR-125b-5p* (figured in bold in the table). In Supplemental Table S1, we present 17 genes that are potentially regulated by more than 10 miRNAs from the list in Table 4, to identify, by another reasoning line, connections between miRNA-regulated genes and endometriosis pathophysiology. As shown in the table, the deregulation of any of these genes was moderate, except for *PTGS1* (9-fold), *SCD* (2.5-fold), *MKI67* (2.1-fold) and *FASN* (1.9-fold), suggesting that for those, one way of modifying their level of expression in the endometriotic lesion is the biosynthesis of specific miRNAs. Interestingly, the genes regulated by ten or more miRNAs (*p* = 0.003) appear to be biologically connected, when agnostically analyzed using the String online tool (https://string-db.org accessed on 1 June 2020). The network composed of these genes is depicted in Supplemental Figure S2, with enrichment in specific pathways, in particular associated with the cellular response to external stimuli.

### 3.3. Quantitative Evaluation of Eight miRNAs in Tissue and Plasma

**Figure 1:**
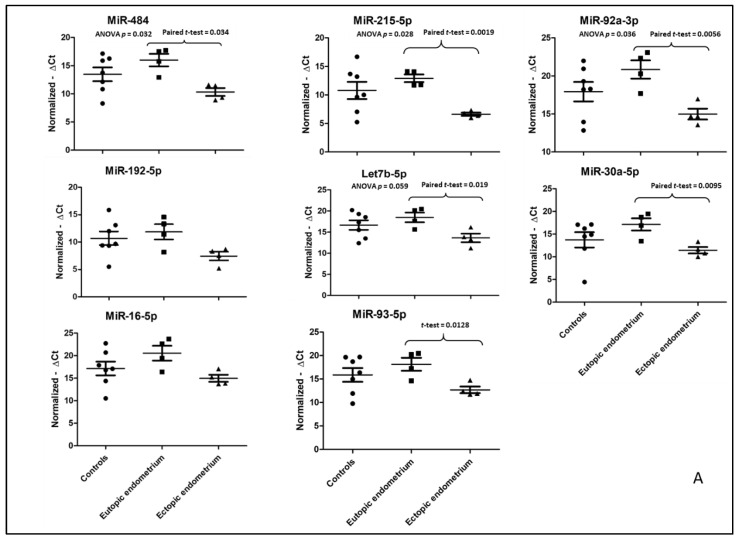

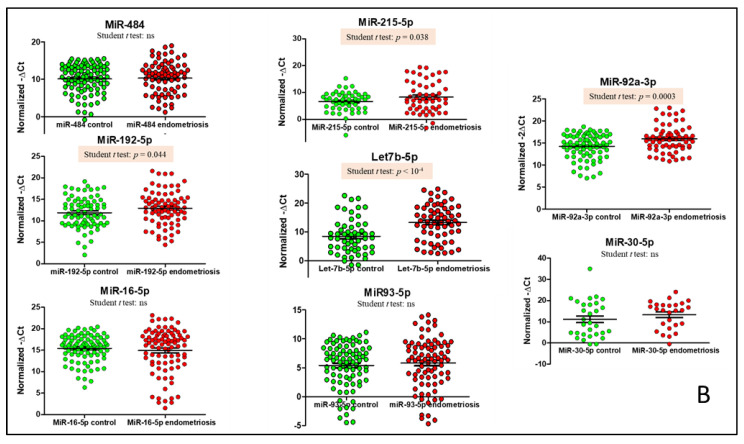
(**A**) Differentially expressed miRNAs in ovarian endometrioma, the eutopic endometrium and normal endometrium. OMA: endometrioma; EE: eutopic endometrium. *n* = 4 patients with endometrioma (providing OMA and matched EE). *n* = 7 controls (providing normal endometrium). Expression was based on qPCR quantification. *p*-values are based on ANOVA and Fisher test, followed by a Student *t*-test for comparing Ectopic Endometrium (EE) versus ovarian endometriotic lesion (OMA) from the same patient. (**B**) Differentially expressed miRNAs in the plasma of patients with ovarian endometrioma (red) and controls (green). *N* = 93 patients with endometrioma (endo). *N* = 95 controls (only part of them were detectable for miR-30-5p). Expression was based on qPCR quantification. *p*-values are based on Student’s *t* test.

The expression of eight miRNAs (*miR-484*, *miR-192-5p*, *miR-16-5p*, *miR-215-5p*, *miR-93-5p*, *miR-92a-3p*, *miR-30a-5p* and *let7b-5p*) was analyzed by qPCR in tissue samples of the ectopic endometrium (from surgically removed OMA) and the eutopic endometrium (from endometrial biopsies) in four OMA patients and compared with samples of normal endometrium in eight controls. We initially chose the top-10 miRNAs (i.e., with the most significant *p*-values according to bioinformatics; see Table 4) but had to eliminate several of them because of technical difficulties in the design of the primers and/or financial constraints. Finally, we selected the eight miRNAs shown in Figure 1A.

Quantifications of the PCR products were normalized by the expression of two miRNAs as controls, *miR-545-3p* and *miR-519e-5p*, that remained unchanged in endometriosis and had constant expression in our experiments. We observed a significant downregulation of *miR-484*, *miR-192-5p*, *miR-215-5p* and *let7b-5p* when comparing the ectopic endometrium (OMA) to the eutopic endometrium. For the other miRNAs, there was a tendency towards decreased levels, but that did not reach statistical significance (*p*-values ranging between 0.05 and 0.08), due to the small number of samples used for this experiment. Then, we quantified the eight miRNAs in the plasmas of 188 patients (93 with OMA and 95 controls) by qPCR (Figure 1B). *Let-7p-5p* was the most differentially expressed in the plasma (almost 30-fold) (*p* < 0.001). We noted a fourth miRNA being modified in the plasma of endometriotic patients: *miR-92a-3p* with an induction ratio of 4-fold (*p* < 0.01) (Figure 1B).

### 3.4. Correlation between miRNA Expression, Phases of the Menstrual Cycle and Clinical Symptoms

**Figure 2:**
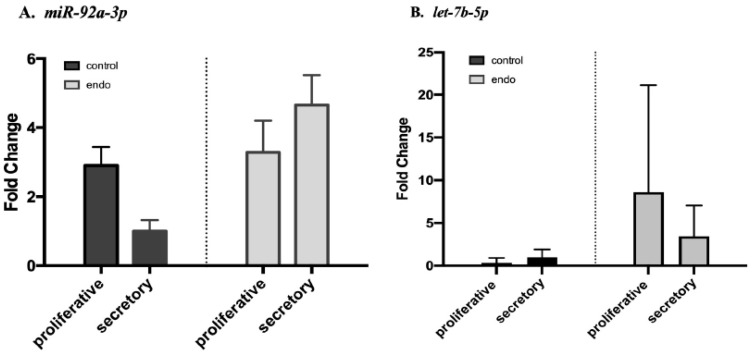
The effect of the menstrual cycle on *miR-92a-3p* and *let-7b-5p* levels of expression in plasma samples from the endometriosis and control groups. Endo: endometriosis group (*n* = 93). Control: healthy patients (*n* = 95). *p*-values are based on analysis by variances of the ANOVA test. (**A**) *miR-92a-3p*. ANOVA *p* = 0.003, interaction effect *p* = 0.032, post-hoc test (SNK) for endometriosis vs. controls *p* = 0.012. (**B**) *let-7b-5p*. ANOVA *p* = 0.045, interaction effect *p* = 0.253, post-hoc test (SNK) for endometriosis vs. controls *p* = 0.013.

Figure 2 shows the differential expression of *miR-92a-3p* and *let7b-5p* in the endometriosis and control groups according to the phase of the menstrual cycle.

For *miR-92a-3p* (Figure 2A), we observed a paradoxical effect of the menstrual cycle: *miR-92a-3p* was more upregulated in the secretory phase than in the proliferative phase among the patients with endometriosis, whereas the reverse was observed among the controls (ANOVA *p* = 0.003, interactive effect *p* = 0.032). Regarding *let7b-5p* (Figure 2B), there was a difference according to the phase of menstrual cycle (ANOVA *p* = 0.045), but no interaction effects between the two factors: disease and phase (*p* = 0.253). We analyzed the eight miRNAs tested with the detailed clinical phenotypes of the patients. We found a significant correlation between their plasma level of expression and several clinical features characterizing endometriosis (Supplemental Figure S2A). For instance, *miR-30a-5p* appeared to be strongly correlated with infertility (r = −0.43, *p* = 0.001) (supplemental Figure S2A). *miR-192-5p* had a correlation with the intensity of dysmenorrhea as evaluated with a visual analogue scale (r = −0.2, *p* = 0.018) (supplemental Figure S2B) and *miR-215-5p* with the intensity of the chronic pelvic pain (r = 0.25, *p* = 0.01) (Supplemental Figure S2C).

### 3.5. miRNAs Signature of Endometriosis

**Figure 3:**
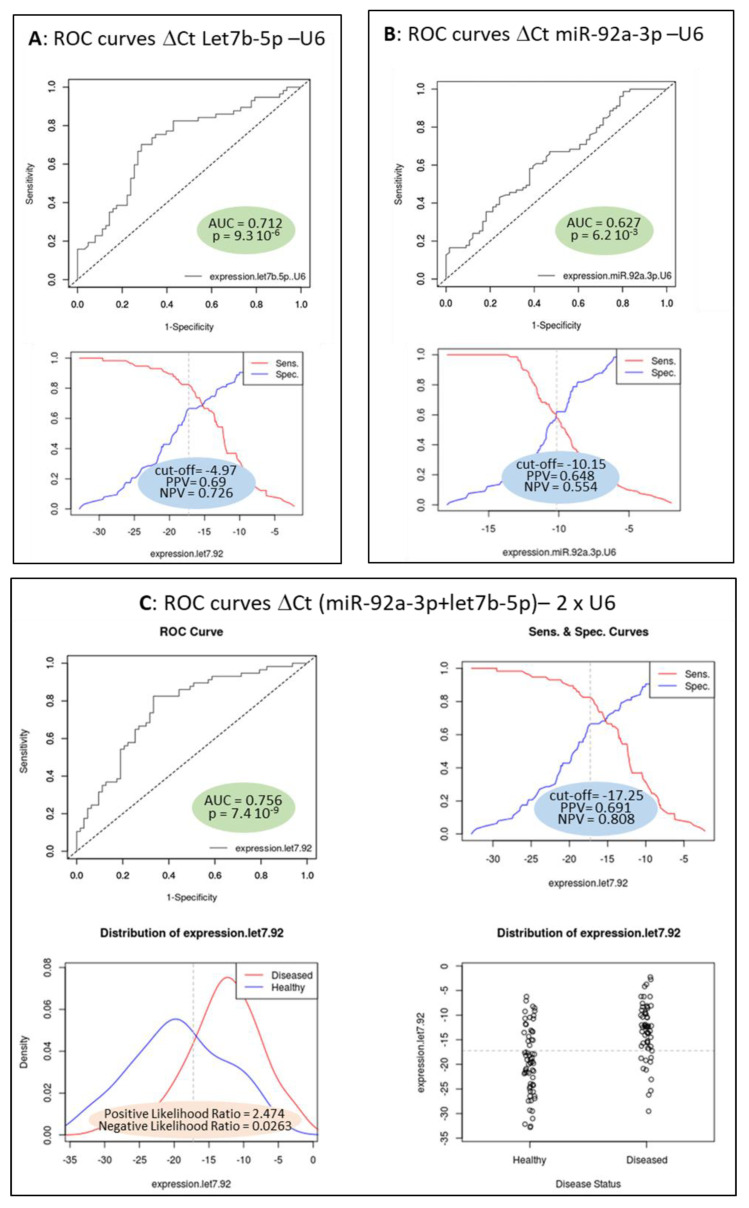
ROC curves for plasmatic levels of *let-7b-5p* (**A**), *miR-92a-3p* (**B**) and the combination of both, called in the figure “expression.let7.92” (in every case, the Ct of the miRNA of interest – Ct of U6 taken as reference, or the sum of both from the RT-qPCR experiments) (**C**) for discriminating endometriosis. *n* = 93 in endometriosis group and *n* = 95 in control group.

Finally, we attempted to use the plasmatic quantification of selected miRNA as a diagnostic tool for endometriosis. From this point of view, we constructed Receiver Operating Characteristic (ROC) curves for a single miRNA or a combination of miRNAs. Figure 3 shows ROC curves for *let7b-5p*, *miR-92a-3p* and a combination of the two, respectively. Area Under the Curve (AUC) for *let-7b-5p* plus *miR-92a-3p* reached 0.73 with a 95% confidence interval between 0.64 and 0.82.

The Areas Under the Curve (AUC) value for *let-7b-5p* was 0.712 (95% CI: 0.619–0.805). The AUC value for *miR-92a-3p* was 0.627 (95% CI: 0.536–0.718). The combination of plasmatic *let-7b-5p* and *miR-92a-3p* revealed the highest AUC value: 0.756 (95% CI: 0.66–0.84). The Cut-off value, the positive predictive values and the negative positive values for these cut-offs were presented in every situation. In the third case, we additionally presented the statistical reliability from the positive and negative likelihood ratios. All the data were calculated using easyROC [21].

### 3.6. KIAA1324, a Potential Target Gene for miRNAs in Endometriosis

Out of the eight miRNAs identified by computational tools, three miRNAs, *let-7b-5p*, *miR-92a-3p*, and with a lesser score *miR-93-5p*, potentially targeted *KIAA1324* (also known as *EIG121* for Estrogen Induced Gene 121), an estrogen-responsive gene and one of the most downregulated genes in OMA. *KIAA1324* is repressed more than 120-fold and has an induction ratio of 0.0084 in OMA, when compared with the eutopic endometrium [11].

**Figure 4:**
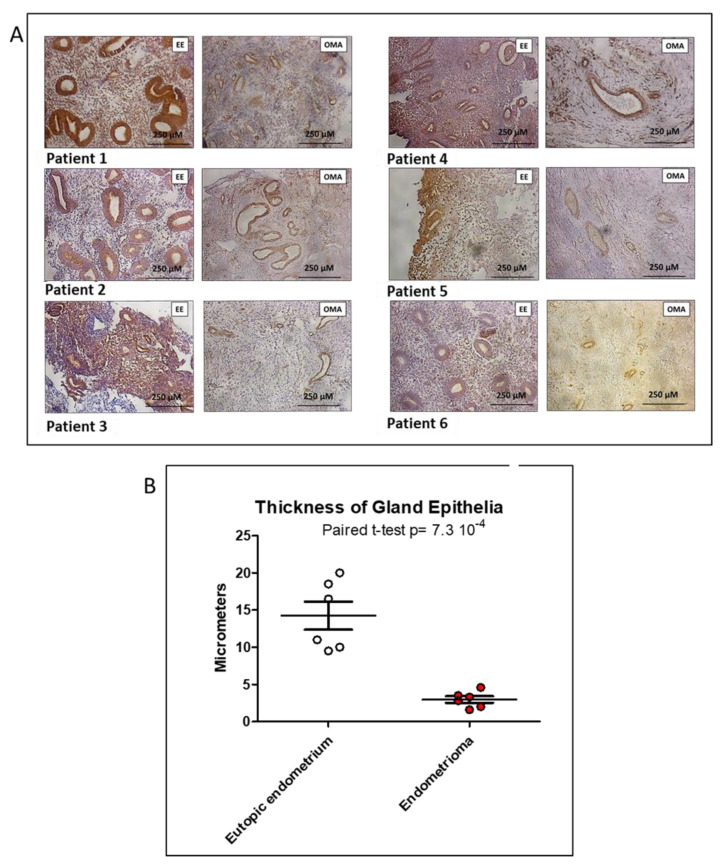
Immunohistochemistry staining of KIAA1324 in ovarian endometrioma and eutopic endometrium (**A**) and quantification by ImageJ (**B**). *n* = 6 patients with endometriosis. OMA: endometrioma; EE: eutopic endometrium. We used a designed antibody against the COOH-terminal peptide of KIAA1324 (1:100).

Immunohistochemistry experiments were carried out on six patients (Table 3) and the results are presented in Figure 4A. They revealed a moderate-to-intense staining for KIAA1324 in the eutopic endometrium and a faint-to-moderate staining in the ectopic endometrium (OMA) for more than half of the samples, which is concordant with the transcriptomic data. In addition, we could observe a systematic decrease in the thickness of the glandular epithelium (Figure 4B); in this case, this was not influenced by the hormonal status, since the same patients were always considered.

**Figure 5:**
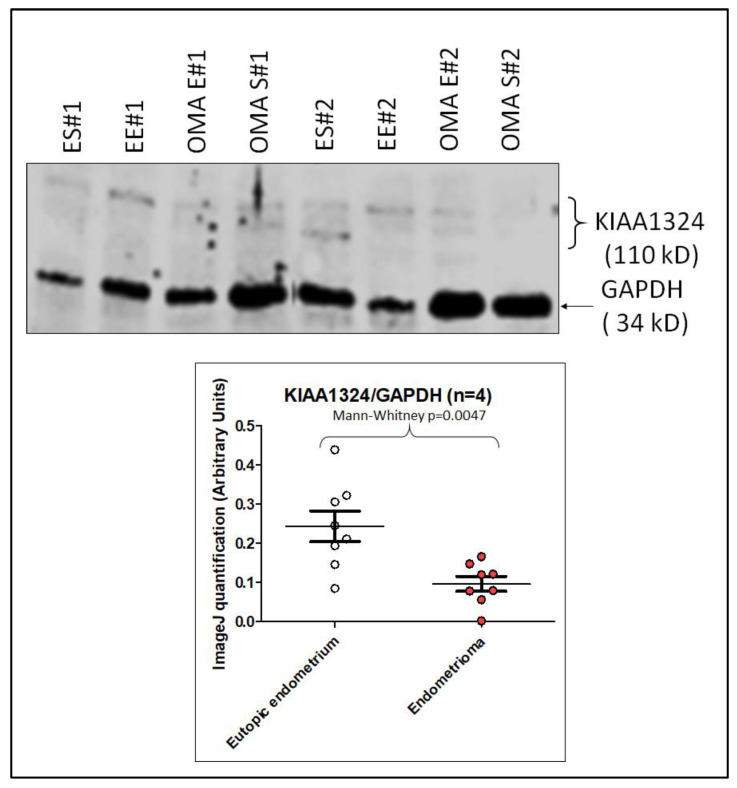
Western blot analysis with two representative patients (denoted as #1 and #2). The antibody against the COOH-terminal peptide of KIAA1324 was used at a concentration of 1:100. The lower part of the figure is a quantitative analysis of the WB using the 4 patients (only 2 are shown in the Western blot) after densitometry analysis by ImageJ. On the WB there are four lanes for each patient: eutopic epithelium, eutopic stroma, lesion epithelium and lesion stroma. Hence the 8 dots presented in the quantification. We never found any significant differences in behaviors of expression between the stromal and epithelial cells, and therefore we analyzed them together. Thus, the four patients led to 8 dots (4 + 4) for the eutopic endometrium and 8 dots for the ectopic endometrium (endometrioma lesion, 4 + 4). ES = Isolated Endometrial Stroma, EE = Isolated Endometrial Epithelium, OMA E = Isolated Endometrioma Epithelium, OMA S = Isolated Endometrioma Stroma.

In addition, we performed a Western blot analysis on four patients using separately purified epithelial and stromal cells. The results are presented in Figure 5.

In vitro validation of the miRNAs’ effect on this target gene was performed on primary cell cultures treated with estrogens. These experiments were conducted on 10 patients, from which a culture of epithelial and stromal cells has been obtained. The clinical characteristics of the patients are presented in Table 3.

**Figure 6:**
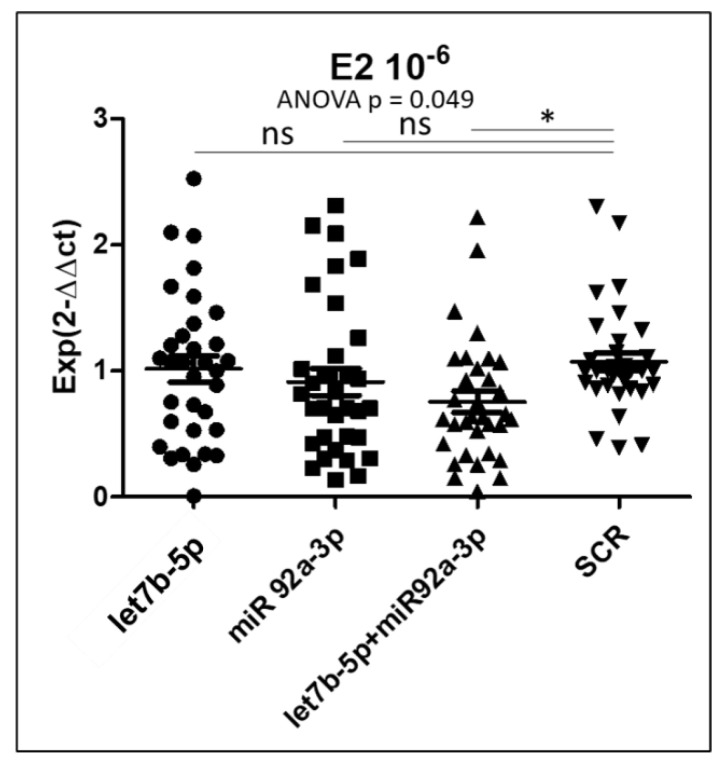
Effects of *let-7b-5p*, *miR-92a-3p* and both of them on *KIAA1324* expression in human endometrial cells transfected with miRNAs mimics and treated with 17β-estradiol. The *KIAA1324* level of expression was measured by qPCR. E_2_ = estradiol. The values of the transcript molecules were normalized to the values of scrambled (SCR) miRNA (*n* = 10 patients). For the post-hoc *t*-test, following ANOVA: *, *p* < 0.05.

Then the cells were treated either with specific siRNAs (mimics of the miRNAs) or scrambled siRNAs. In addition, we treated the samples with 17b-estradiol according to a gradient of concentration (0 M, 10^−8^ M and 10^−6^ M). Following the normalization, a three-factor ANOVA (factors being patient, estrogen treatment and cell type) was performed and was found to be significant (*p* = 6.1 × 10^−4^). Detailed analysis revealed that the patient effect and the cell-type were not significant (*p* = 0.123 and *p* = 0.577, respectively), while the estrogen treatment was significant (*p* = 5.7 × 10^−4^), as were the siRNA effects (*p* = 1.7 × 10^−2^) (Figure 6).

Starting from these preliminary analyses, ANOVA was carried out to test the siRNA effects at three possible doses of estrogens and only the highest concentration (10^−6^) gave a significant *p* value (*p* = 0.049). A Dunnett post-hoc test revealed that only the combination of both siRNA was able to reduce KIAA1324 expression by ~40%.

As mentioned, the absence of differences when analyzing the stromal or epithelial endometrial cells separately was consistent with the staining in Figure 4 and the Western blot in Figure 5, which showed a staining of both the epithelium and the stroma, in this case of endometriotic women (previously published staining data on KIAAim24 showed most or all of the staining on epithelial cells, but it was done only on control -non endometriotic women).

## 4. Discussion

To identify miRNAs potentially involved in OMA, one of the main subtypes of the disease, we developed an original and unbiased approach based on bioinformatic analyses of two large databases: miRWalk2.0, a comprehensive atlas of miRNA-target interactions, and a highly validated transcriptomic dataset of OMA. This strategy was legitimated by the wide range of non-reproducible sets of miRNAs proposed as endometriosis markers [10,22]. Using this method, we expected to detect miRNAs that do have an effective effect on gene expression. We identified putative regulating miRNAs and validated their deregulation in a large number of women afflicted with endometriosis, both in the tissue and in the plasma. Our study population included 216 individuals who were operated on for benign conditions, including endometriosis, which allowed us to distinguish unambiguously the cases (with OMA) from the controls (without endometriosis). In the future, using more individuals with the same approach could help to identify more miRNAs with a significantly different level of expression between the controls and endometriotic women. In addition, the 16 miRNA that were not studied further could be analyzed in future experiments.

To the best of our knowledge, no correlation has been reported between the miRNAs we studied and the main indications for surgery in the control group (i.e., infertility and benign ovarian cysts). Here we studied miRNA levels both in the endometrial tissue and lesion and in the plasma. In this case miRNAs are considered as putative biomarkers, which is an important challenge for a disease where the average length from the declaration of symptoms to diagnosis is estimated at seven years.

Interestingly, some of the miRNAs found to be increased in plasma correlated with clinical symptoms, especially *miR-192-5p* with dysmenorrhea, *miR-215-5p* with pelvic pain, and *miR-30-5p* with infertility. It seems promising to follow this direction and study a larger population of well-characterized women, afflicted with various subtypes of the disease, such as deep endometriosis or superficial endometriosis, and various severity of painful symptoms and infertility. As preliminarily shown by ROC curves, plasmatic levels of *miR-92a-3p* and *let-7b-5p* might be contributive to discriminate the patients with endometriosis from healthy subjects and could serve as the first step for the development of a non-invasive test to diagnose endometriosis earlier than it is diagnosed today [23]. Together with other robust approaches attempting to identify and validate valid miRNAs in independent cohorts [23,24], our approach based on potential targeting of mRNA by miRNAs is complementary and could help to define an ultimately efficient panel of miRNA markers. In addition, it is obviously of great interest to identify miRNAs that can predict endometriosis subtypes with accuracy, or that vary during the natural history of the disease and may be used in follow-up. In view of our results, quantification of circulating miRNAs is certainly a promising diagnostic tool, but we must not forget that miRNA expression remains vulnerable to the constraints of blood sampling, time and menstrual cycle variations for some patients [22]. In addition, one must keep in mind that current techniques only extract a limited fraction of the total miRNAs in circulation, probably a small percentage, which could bias the results [25,26].

Our findings lead us to focus on *KIAA1324* (*EIG121* or *ELAPOR1*), which is one of the most downregulated genes in OMA, according to the transcriptomic data. This gene, whose expression is modulated by estrogens, encodes a transmembrane protein expressed in the normal endometrium. It could have a role in the cellular response to stress [12] and act as a tumor suppressor gene, as it has been found to be strongly deregulated in endometrial serous carcinoma [16,27]. The possible link of endometriosis with autophagy is now relatively well documented [28,29]. The massive downregulation of *KIAA1324* in OMA is an indication that the endometriosis lesions could have an altered fitness, and is thus better for the patient. This led to a discussion of the advantages or drawbacks of autophagy in the context of this disease [30]. In the literature, *KIAA1324* has essentially be detected in endometrial glands [16], while we found it in both the stromal and epithelial parts of the endometrium and of the OMA, in such a way that the difference of behavior was not obvious and allowed us to pool the mimics of miRNAs in both cell types. This observation could be related to the fact that contrary to the Deng paper and the Human Protein Atlas database, our samples are from women that were all endometriotic, and there could be a specific stromal labeling in these patients, contrary to the control patients. In concordance with histology data, we found an increased labeling in the luminal part of the glands in the histology. The gene was considerably increased in tumors with excess estrogen [16], while it was decreased in endometriosis, a fact of which we provide some mechanistical insights in this paper. This difference could be a reflection of the non-tumoral characteristic of endometriosis.

We systematically found (be it at the mRNA level, or at the protein level by IHC or WB) that the gene and the protein were downregulated in the lesion compared with the endometrium. The impact of miRNAs in downregulating *KIAA1324* appeared real but limited to ~40% decrease and only in the presence of estrogens at high doses (10^−6^). In summary, the regulation of *KIAA1324* is quite complex and depends upon a transcription factor network (via ESR1 or ESR2, in particular) but also through a complex of miRNAs present in the cells. *KIAA1324* is targeted by three miRNAs predicted to be increased in endometriosis, especially by *let-7b-5p* and *miR-92a-3p*, the two miRNAs we found to be significantly overexpressed in the tissue and plasma of endometriotic patients. *Let-7b* has been previously reported in endometriosis by others [31,32] and *miR-92a* could promote progesterone resistance in endometriosis [33], which reinforces the validity of our results. Immunohistochemistry showed very low levels of the KIAA1324 protein in OMA, when compared to the eutopic endometrium. Transfection experiments of miRNAs mimics in primary cultured endometrial cells clearly demonstrated that *miR-92a-3p* and *let-7b-5p* may act together to downregulate the expression of *KIAA1324* in the presence of estrogens. *MiR-92a-3p* appeared to be influenced by the menstrual cycle phase, and was strongly overexpressed in the secretory phase. This agrees with our findings that the in vitro expression of *KIAA1324* seems to vary according to the concentration of estradiol (E2); it is strongly repressed when E2-concentration is high (as in the proliferative phase), is unchanged when E2-concentration is intermediate (as in the secretory phase) and is slightly repressed when E2-concentration is low (as during the menses), thus reproducing the variation of estradiol production during the menstrual cycle. Given what we know about the influence of hormone variation during the menstrual cycle and endometriosis development and its symptoms (with a recrudescence of painful symptoms during menses), this is a strong argument in favor of the implication of *KIAA1324* in the pathogenesis of the disease. Nevertheless, further studies are mandatory to determine precisely the exact mechanisms by which *let-7b-5p* and *miR-92a-3p* may partially control the expression of *KIAA1324* and how and when this gene interferes with the development of endometriotic lesions.

In conclusion, our study unveiled a potentially major gene implicated in endometriosis, *KIAA1324*, by means of an unbiased bioinformatic analysis, and highlighted the full potential of circulating miRNAs as an efficient tool for the early diagnosis and follow-up of endometriosis.

## Figures and Tables

**Table 1:** Clinical characteristics of the study population. NRS: Numerical Rating Scale. BMI: body mass index. A *t*-test was used for a comparison of continuous variables and a chi-square test for categorical variables. SD: standard deviation.

	Control Group (*n* = 103)	Endometriosis Group (*n* = 113)	Entire Population (*n* = 216)	*p*-Value
**Age (years, mean ± SD)**	30.6 ± 6.2	31.9 ± 5.1	31.3 ± 5.7	0.094
**BMI (kg/m^2^, mean ± SD)**	22.4 ± 4.0	21.6 ± 4.0	22.0 ± 4.0	0.220
**Smoking, *n* (%)**	39 (38%)	45 (40%)	84 (39%)	0.381
**Cycle phase, *n* (%)**				
Follicular	35 (44%)	34 (50%)	69 (47%)	0.395
Luteal Missing data	44 (56%) 24	34 (50%) 45	78 (53%) 69	
**Ethnicity**				
Caucasian	72 (70%)	84 (74%)	156 (72%)	0.529
African	16 (15%)	9 (8%)	25 (11.6%)	0.121
Asian	3 (3%)	1 (1%)	4 (2%)	0.312
**Previous surgery for endometriosis, *n* (%)**	-	18 (16%)	-	-
**Familial history of endometriosis, *n* (%)**	3 (3%)	8 (7%)	11 (5%)	0.120
**Hormonal treatment, *n* (%)** Missing data	22 (58%) 65	5 (19%) 87	27 (42%) 152	0.002
**Pain symptoms, *n* (%)**				
Dysmenorrhea, *n* (%)	56 (54%)	75 (66%)	131 (61%)	0.003
Primary	35 (34%)	50 (44%)	85 (39%)	0.0001
Secondary	21 (20%)	25 (22%)	46 (21%)	0.498
NRS dysmenorrhea (mean ± SD)	3.9 ± 3.3	5.6 ± 3.2	4.7 ± 3.4	0.0007
NRS dyspareunia (mean ± SD)	1.6 ± 2.8	1.5 ± 3.3	2.3 ± 3.1	0.0097
NRS pelvic pain (mean ± SD)	1.6 ± 2.7	1.6 ± 3	1.9 ± 2.9	0.159
**Infertility, *n* (%)**	43 (42%)	33 (29%)	76 (35%)	0.139
Primary	25 (24%)	26 (23%)	51 (24%)	0.869
Secondary	18 (17%)	7 (6%)	25 (12%)	0.018

**Table 2:** Clinical characteristics of the study population selected for immunohistochemistry assays: *n* = 6 patients with endometriosis (OMA). NRS: Numerical Rating Scale. BMI: body mass index. SD: standard deviation.

**Age (years, mean** **± SD)**	35.8 ± 3.5
**BMI (kg/m^2^, mean** **± SD)**	22.6 ± 1.2
**Smoking, *n* (%)**	2 (33%)
**Cycle phase, *n* (%)**	
Follicular	3 (50%)
Luteal Missing data	3 (50%) 0
**Ethnicity**	
Caucasian	5 (83%)
African	1 (17%)
Asian	0 (0%)
**Previous surgery for endometriosis, *n* (%)**	1 (17%)
**Familial history of endometriosis, *n* (%)**	1 (17%)
**Hormonal treatment, *n* (%)** Missing data	4 (67%) 0
**Pain symptoms, *n* (%)**	
Dysmenorrhea, *n* (%)	4 (67%)
Primary	3 (50%)
Secondary	1 (17%)
NRS dysmenorrhea (mean ± SD)	5. ± 2.6
NRS dyspareunia (mean ± SD)	2.3 ± 2.3
NRS pelvic pain (mean ± SD)	2.6 ± 2.1
**Infertility, *n* (%)**	2 (33%)
Primary	2 (33%)
Secondary	0 (0%)

**Table 3:** Clinical characteristics of the study population selected for transfection experiments: *n* = 10 patients with endometriosis:(OMA). NRS: Numerical Rating Scale. BMI: body mass index. SD: standard deviation.

**Age (years, mean ± SD)**	34.2 ± 3.5
**BMI (kg/m^2^, mean ± SD)**	22.4 ± 1.6
**Smoking, *n* (%)**	3 (30%)
**Cycle phase, *n* (%)**	
Follicular	5 (50%)
Luteal Missing data	5 (50%) 0
**Ethnicity**	
Caucasian	8 (80%)
African	1 (10%)
Asian	1 (10%)
**Previous surgery for endometriosis, *n* (%)**	2 (20%)
**Familial history of endometriosis, *n* (%)**	1 (10%)
**Hormonal treatment, *n* (%)** Missing data	7 (70%) 0
**Pain symptoms, *n* (%)**	
Dysmenorrhea, *n* (%)	6 (60%)
Primary	5 (50%)
Secondary	1 (10%)
NRS dysmenorrhea (mean ± SD)	5.4. ± 2.7
NRS dyspareunia (mean ± SD)	2.3 ± 1.9
NRS pelvic pain (mean ± SD)	2.7 ± 2
**Infertility, *n* (%)**	4 (40%)
Primary	3 (30%)
Secondary	1 (10%)

**Table 4:** List of putative miRNAs regulating genes differentially expressed in ovarian endometrioma, as predicted by bioinformatic analysis.

miRNA	Number of Genes Regulated by miRNA			*p*-Value ^‡^	Reference
	Downregulated ^†^	Unchanged ^†^	Upregulated ^†^		
*miR-484*	49	708	22	4.4 × 10^−18^	
*miR-92a-3p*	63	848	38	6.4 × 10^−17^	
*miR-192-5p*	154	727	53	2.6 × 10^−16^	
*miR-16-5p* *	100	1003	48	3.5 × 10^−16^	Braza-Boïls, 2015 [17]
*miR-615-3p*	57	789	36	7.2 × 10^−16^	
*miR-215-5p*	123	543	37	2.1 × 10^−15^	
*let-7b-5p* *	80	819	33	2.1 × 10^−15^	Papari et al., 2020 [18]
*miR-193b-3p*	75	671	27	2.3 × 10^−12^	
*miR-30a-5p*	53	376	11	5.2 × 10^−9^	
*miR-186-5p*	45	467	22	3.5 × 10^−8^	
*miR-877-3p*	27	357	15	8.0 × 10^−8^	
*miR-335-5p*	306	1802	348	8.8 × 10^−8^	
*miR-155-5p*	88	589	39	1.6 × 10^−7^	
*miR-320a*	36	418	0	3.9 × 10^−7^	
*miR-93-5p* *	38	373	16	4.9 × 10^−7^	Lv et al., 2015 [19]
*let-7e-5p*	18	269	10	6.7 × 10^−7^	
*miR-149-5p* *	17	276	13	1.7 × 10^−6^	Braza-Boïls, 2015 [17]
*miR-744-5p*	20	321	20	4.9 × 10^−6^	
*miR-222-3p* *	17	259	13	1.1 × 10^−5^	Ramon et al., 2011 [20]
*miR-92b-3p*	11	200	8	1.2 × 10^−5^	
*miR-324-3p*	12	189	6	1.5 × 10^−5^	
*miR-93-3p* *	9	180	7	1.9 × 10^−5^	Lv et al., 2015 [19]
*miR-1* *	97	681	59	3.4 × 10^−5^	Ohlsson Teague et al., 2009 [8]
*miR-125b-5p*	39	237	12	6.3 × 10^−5^	

List of miRNAs presenting a significant bias between the number of genes up- or downregulated. * In bold, miRNA previously reported to be associated with endometriosis according to the published literature. ^†^ Downregulated: defined by an induction ratio <0.5 in the ectopic endometrium when compared to the eutopic endometrium; Upregulated: defined by an induction ratio >2.0; unchanged: induction ratio between 0.5 and 2.0. ^‡^ Chi-square test when compared with the whole genome. Bonferroni correction was used for multiple testing.

## Data Availability

All the data will be freely available upon request.

## Supplementary Materials

The following supporting information can be downloaded at: https://www.mdpi.com/article/10.3390/biomedicines10092065/s1, Table S1: List of the genes under the control of ten or more miRNAs.; Figures S1: Immunohistochemistry of the pancreas with the KIAA1324 antibody, Figure S2: Network analysis of the genes regulated by ten or more of the identified miRNAs, and S3: Correlation between clinical features and miRNA plasmatic level of expression in women with and without endometriosis.
