# Supplementary material for: Unbiased In Silico Analysis of Gene Expression Pinpoints Circulating miRNAs Targeting KIAA1324, a New Gene Drastically Downregulated in Ovarian Endometriosis
Source: Biomedicines. 2022 Aug 24;10(9):2065. doi: 10.3390/biomedicines10092065 (PMC9495942; doi:10.3390/biomedicines10092065)
Supplement: Supplementary file 1 [file biomedicines-10-02065-s001.zip › biomedicines-1845100-supplementary.pdf]

biomedicines

Supplemental Materials

Table S1. List of the genes under the control of ten or more miRNAs.

Number of regulating miRNAs	Gene symbol	Gene name	Induction ratio*
14	*HSPA1B*	heat shock protein family A (Hsp70) member 1B	1.14
13	*AGO1*	argonaute 1, RISC catalytic component	1.37
12	*EEF2*	eukaryotic translation elongation factor 2	1.13
	EEF1A1	eukaryotic translation elongation factor 1 alpha 1	1.02
	WNK1	WNK lysine deficient protein kinase 1	0.73
	FLNA	filamin A, alpha	1.46
	PTGS1	prostaglandin-endoperoxide synthase 1 (prostaglandin G/H synthase and cyclooxygenase)	0.11
	FASN	fatty acid synthase	0.53
11	*SCD*	stearoyl-CoA desaturase (delta-9-desaturase)	0.40
	NCAPD2	non-SMC condensing I complex subunit D2	0.88
	PRRC2A	proline-rich coiled-coil 2A	0.88
	HUWE1	HECT, UBA and WWE domain containing 1, E3 ubiquitin protein ligase	0.79
	IRS4	insulin receptor substrate 4	0.76
10	*BAG6*	BCL2 associated athanogene 6	0.99
	PRPF8	pre-mRNA processing factor 8	0.78
	GAPDH	glyceraldehyde-3-phosphate dehydrogenase	0.65
	MK167	marker of proliferation Ki-67	0.47

*Level of expression in ectopic endometrium as compared to eutopic endometrium (data from Borghese et al. 2008).

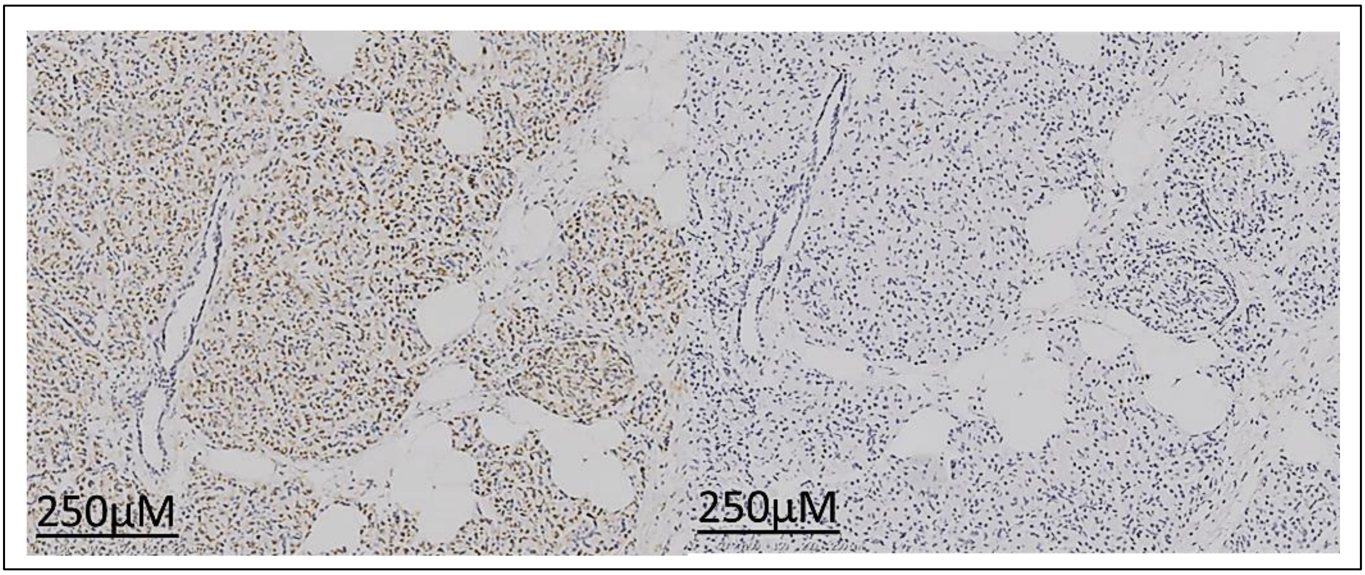

Figure S1. Immunohistochemistry of the pancreas with the KIAA1324 antibody (left) or with IGG (right, negative control). Dilution at 1:100.

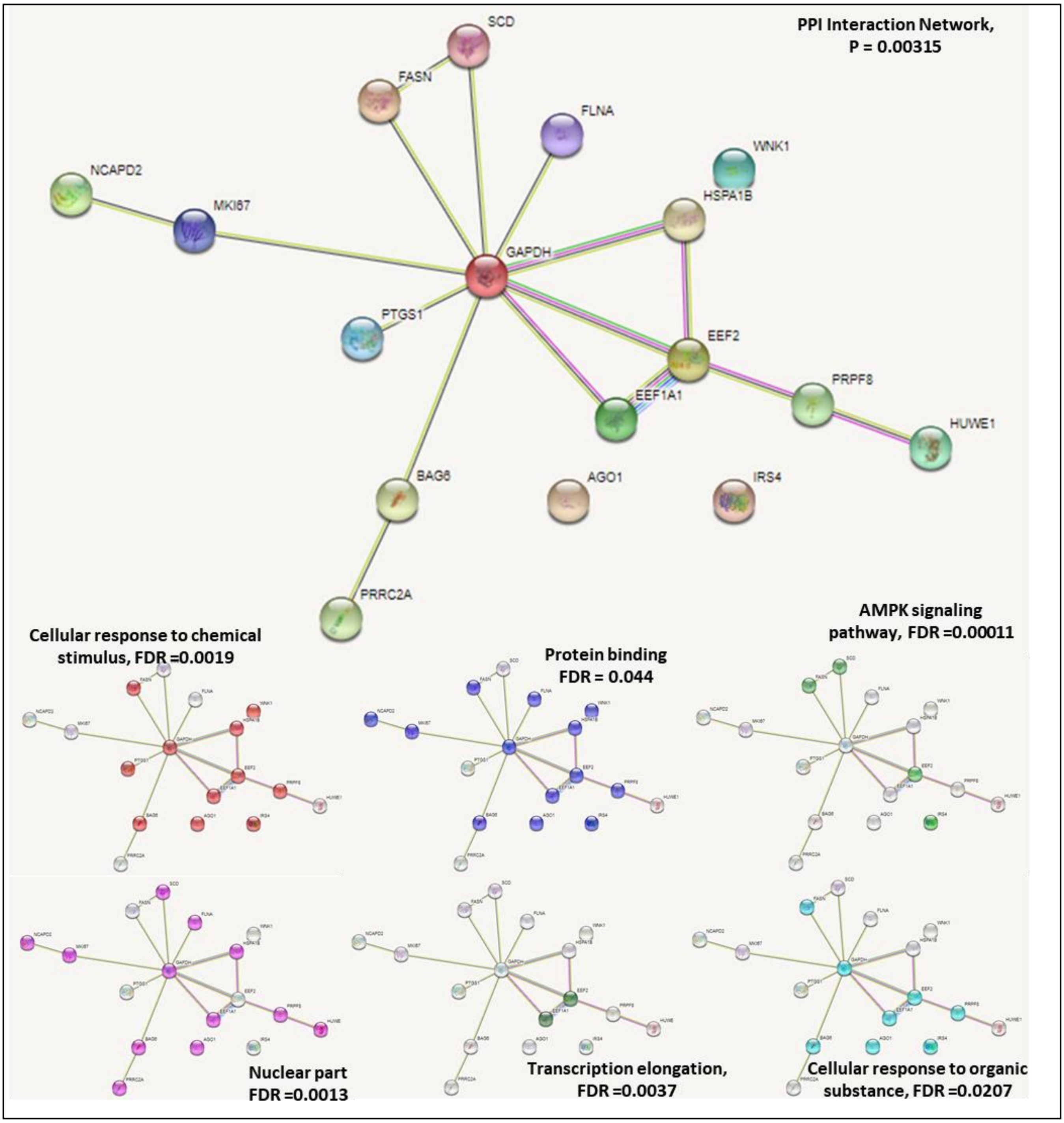

Figure S2. Network analysis of the genes regulated by ten or more of the identified miRNAs. Gene interaction enrichment is significant ($p < 0.001$). In the lower part, the same network is colored with the specific factor specific of each clustering, and the statistical value assigned to each keyword. The colors characterize the genes involved in specific process, and the FDR is the corrected statistical value. For instance, the pink color labels proteins that are localized inside the nucleus and that are present at a statistically increased frequency compare to mere chance.

(A)

	Age	Dysmenorrhea	Dyspareunia	Pelvic Pain	Phases of the Menstrual Cycle	Tobacco	Family History of Endometriosis	CA-125	Infertility
**miR-484**	ns	ns	ns	ns	ns	ns	ns	ns	ns
**miR-192-5p**	ns	r=0.20/p=0.018	ns	ns	ns	r=0.15/p=0.063	ns	ns	ns
**miR-16-5p**	ns	ns	ns	r=0.13/p=0.079	ns	ns	ns	ns	ns
**miR-215-5p**	ns	ns	ns	r=0.25/p=0.01	ns	ns	ns	ns	ns
**let-7b-5p**	ns	ns	ns	ns	ns	ns	ns	ns	ns
**miR-93-5p**	ns	ns	ns	ns	ns	ns	ns	ns	ns
**miR-92a-3p**	ns	ns	ns	ns	ns	ns	ns	ns	ns
**miR-30a-5p**	ns	ns	ns	ns	ns	ns	ns	ns	r=0.43/p=0.001

(B)

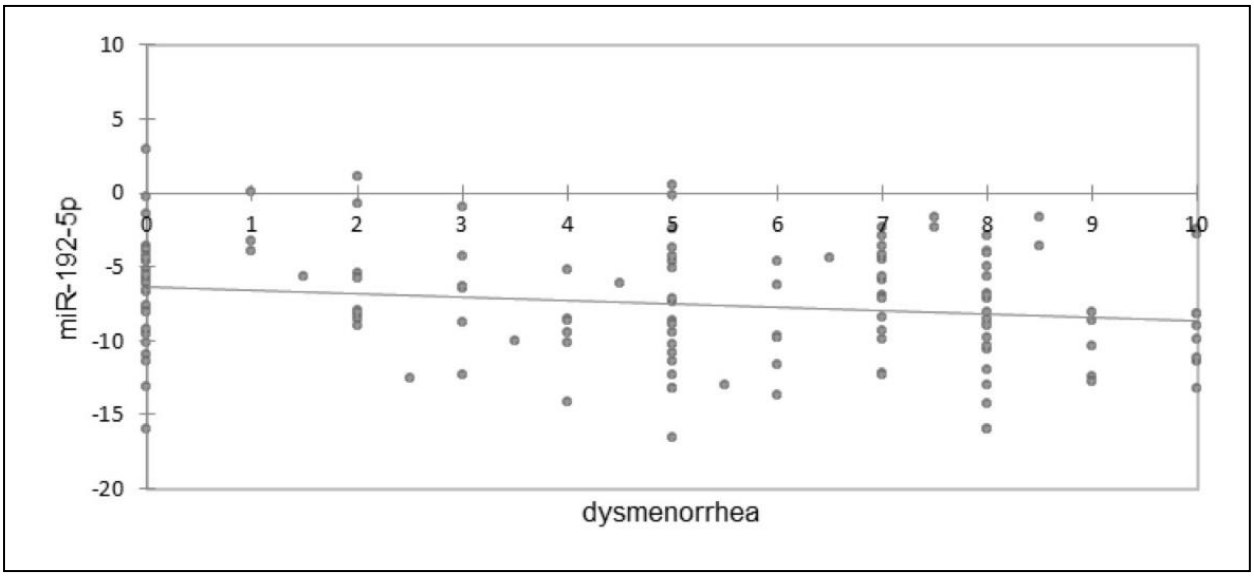

(C)

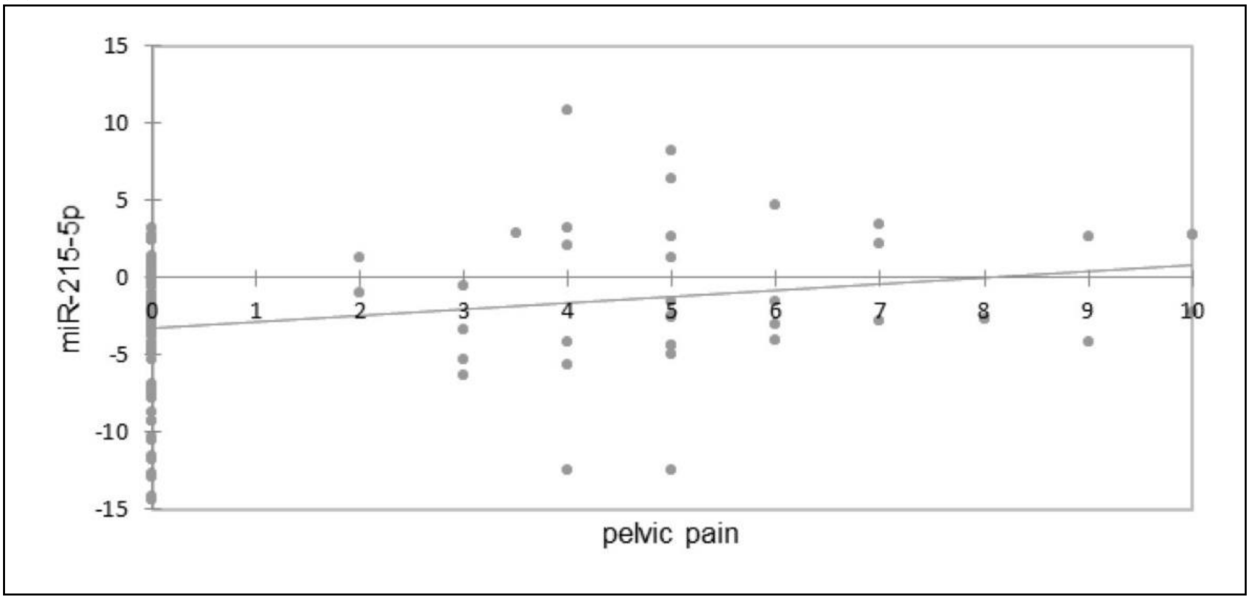

Figure S3. Correlation between clinical features and miRNA plasmatic level of expression in women with and without endometriosis (*N* = 93 and 95, respectively). (**A**) Global correlations with clinical characteristics of endometriosis. Ns = non-significant. (**B**) Scatter plot showing relationship between *miR-192-5p* plasmatic level of expression and the intensity of dysmenorrhea (r = −0.2; *p* = 0.018). (**C**) Scatter plot showing relationship between *miR-215-5p* plasmatic level of expression and the intensity of pelvic pain (r = 0.25; *p* = 0.01).
